# Author Correction: Plant species determine tidal wetland methane response to sea level rise

**DOI:** 10.1038/s41467-023-37364-5

**Published:** 2023-03-22

**Authors:** Peter Mueller, Thomas J. Mozdzer, J. Adam Langley, Lillian R. Aoki, Genevieve L. Noyce, J. Patrick Megonigal

**Affiliations:** 1 Smithsonian Environmental Research Center, Edgewater, MD 21037 USA; 2 Institute of Soil Science, Center for Earth System Research and Sustainability (CEN), Universität Hamburg, 20146 Hamburg, Germany; 3 Department of Biology, Bryn Mawr College, Bryn Mawr, PA 19010 USA; 4 Department of Biology, Center for Biodiversity and Ecosystem Stewardship, Villanova University, Villanova, PA 19003 USA; 5 Department of Ecology and Evolutionary Biology, Cornell University, Ithaca, NY 14853 USA

**Keywords:** Carbon cycle, Climate-change ecology

Correction to: *Nature Communications* 10.1038/s41467-020-18763-4, published online 14 October 2020

The original version of this Article contained an error in the caption title of Figure 4, which incorrectly read ‘CH_4_ emissions from field pots.’ The correct version replaces this sentence with ‘CH_4_ emissions from field plots’. This has been corrected in both the PDF and the HTML versions of the Article.

