# Supplementary material for: Plant species determine tidal wetland methane response to sea level rise
Source: Nat Commun. 2020 Oct 14;11:5154. doi: 10.1038/s41467-020-18763-4 (PMC7560622; doi:10.1038/s41467-020-18763-4)
Supplement: Supplementary file 1 — Supplementary Information [file 41467_2020_18763_MOESM1_ESM.pdf]

Supplementary Information

for *Plant species determine tidal wetland methane response to sea level rise*

P. Mueller et al. (2020)

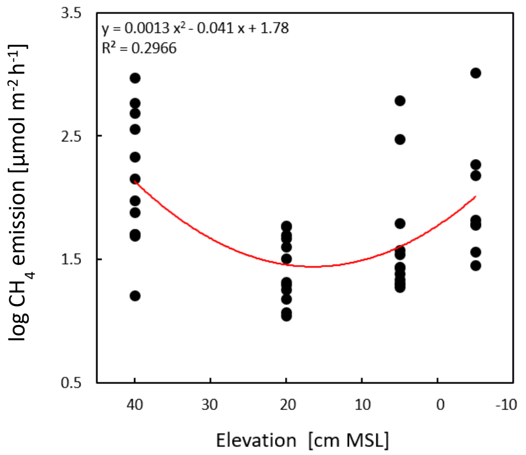

Supplementary Figure 1 Relationship between marsh elevation above mean sea level (MSL) and log CH_4 emissions (n = 44 duplicate mesocosms)

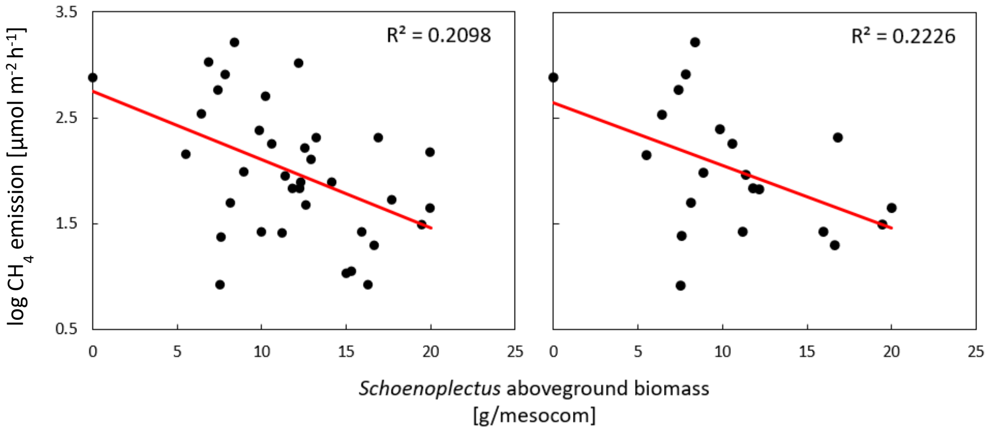

Supplementary Figure 2 Relationship between *Schoenoplectus* aboveground biomass and log CH_4 emissions within elevated CO_2 treatments across all four sea level treatments (left panel; n = 38 single mesocosms) and across across the two highest (least flooded) treatments (+40 cm and +20 cm above sea level; right panel; n = 21 single mesocosms) of Experiment 1.

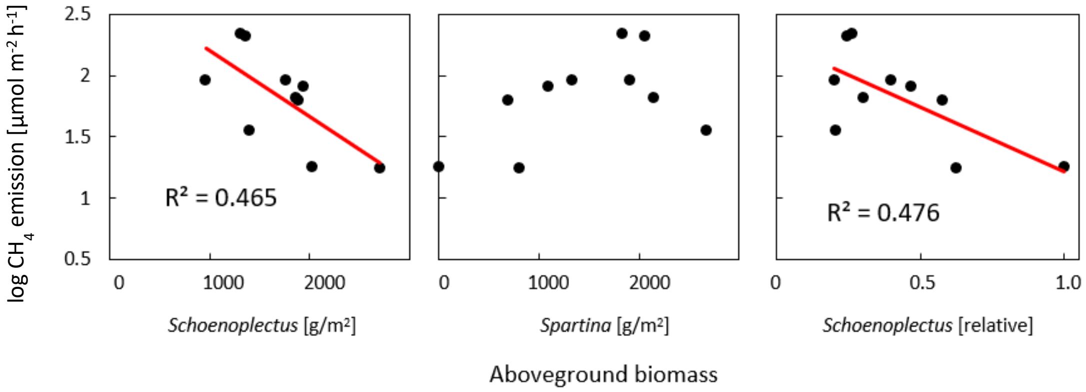

Supplementary Figure 3 Relationships between aboveground biomass and log CH_4 emissions within ambient CO_2 treatments of Experiment 1. Dataset is restricted to the MSL +40 cm treatment (highest elevation). Compare Figure 3 (n = 10 single mesocosms).

Supplementary Table 1 Overview of Pearson correlations between plant biomass parameters and log CH_4 emissions at different sea level treatments in Experiment 1 across all CO_2 and N treatment combinations. Correlations are bold typed at $p \leq 0.1$. Elevated CO_2 and N significantly increased biomass parameters across sea-level treatments (Langley et al. 2013). Significant stimulations within sea-level treatments based on two-way ANOVA (at $p \leq 0.05$; taken from Langley et al. 2013) are indicated as "CO_2" and "N" behind correlation results.

Elevation MSL	40 cm			20 cm			+5 cm			-5 cm		
	Linear r	p-value		Linear r	p-value		Linear r	p-value		Linear r	p-value	
Sc AG	-0.127	0.584	CO_2	-0.106	0.657		-0.343	0.178		-0.117	0.748	N
Sp AG	-0.107	0.645	N	0.112	0.638	N	-0.353	0.164		0.000	1.000	
Total AG	-0.165	0.464	N	0.025	0.918	N	**-0.522**	**0.031**	N	-0.117	0.748	N
Total BG	0.064	0.806		0.162	0.565		-0.209	0.438		0.166	0.587	N, CO_2
%Sp	0.139	0.560		0.062	0.796		-0.323	0.206		0.000	1.000	
AG:BG	-0.294	0.252		0.172	0.539		0.351	0.182		0.215	0.579	

Notes:
Sc AG: Schoenoplectus aboveground biomass
Sp AG: Spartina aboveground biomass
Total AG: total aboveground biomass
Total BG: total belowground biomass
%Sp: percentage Spartina aboveground biomass
AG:BG: ratio of total above vs. belowground biomass

Supplementary Table 2 Overview of Pearson correlations between plant biomass parameters and log CH_4 emissions across all treatment combinations of Experiment 1. Correlations are bold typed at $p \leq 0.1$.

Biomass parameter	Linear r (Pearson)	p-value
N = 82 (total)		
Sc AG	**-0.379**	**0.001**
Sp AG	-0.041	0.741
Total AG	**-0.347**	**0.004**
Total BG	-0.025	0.851
Total	-0.086	0.520
%Sp	0.060	0.631
AG:BG	0.111	0.412
N = 32 (subset)		
Fine roots	-0.232	0.244
Rhizomes	-0.265	0.181
Sc rhizomes	**-0.328**	**0.092**
Sp rhizomes	0.009	0.963
Depth dist. (ß)	-0.139	0.497

Notes:
Sc: Schoenoplectus aboveground biomass
Sp AG: Spartina aboveground biomass
Total AG: total aboveground biomass
Total BG: total belowground biomass
%Sp: percentage Spartina aboveground biomass
AG:BG: ratio of total above vs. belowground biomass
Sc rhizomes: Schoenoplectus rhizome biomass
Sp rhizomes: Spartina rhizome biomass
Depth dist. (ß): Rooting depth distribution (*sensu* Gale and Grigal 1987)

Supplementary Information References

Langley JA, Mozdzer TJ, Shepard KA, Hagerty SB, Megonigal JP (2013) Tidal marsh plant responses to elevated CO2, nitrogen fertilization, and sea level rise. Global Change Biol 19(5):1495–1503

Gale MR, Grigal DF (1987) Vertical root distributions of Northern tree species in relation to successional status. Can J For Res 17:829–834
