# Supplementary material for: Plant species determine tidal wetland methane response to sea level rise
Source: Nat Commun. 2020 Oct 14;11:5154. doi: 10.1038/s41467-020-18763-4 (PMC7560622; doi:10.1038/s41467-020-18763-4)
Supplement: Supplementary file 3 — Reporting Summary [file 41467_2020_18763_MOESM3_ESM.pdf]

nature research

Corresponding author(s): Peter Mueller

Last updated by author(s): Aug 27, 2020

Reporting Summary

Nature Research wishes to improve the reproducibility of the work that we publish. This form provides structure for consistency and transparency in reporting. For further information on Nature Research policies, see Authors & Referees and the Editorial Policy Checklist.

Statistics

For all statistical analyses, confirm that the following items are present in the figure legend, table legend, main text, or Methods section.

n/a	Confirmed	
☐	☒	The exact sample size (*n*) for each experimental group/condition, given as a discrete number and unit of measurement
☐	☒	A statement on whether measurements were taken from distinct samples or whether the same sample was measured repeatedly
☐	☒	The statistical test(s) used AND whether they are one- or two-sided<br>*Only common tests should be described solely by name; describe more complex techniques in the Methods section.*
☐	☒	A description of all covariates tested
☐	☒	A description of any assumptions or corrections, such as tests of normality and adjustment for multiple comparisons
☐	☒	A full description of the statistical parameters including central tendency (e.g. means) or other basic estimates (e.g. regression coefficient) AND variation (e.g. standard deviation) or associated estimates of uncertainty (e.g. confidence intervals)
☐	☒	For null hypothesis testing, the test statistic (e.g. *F*, *t*, *r*) with confidence intervals, effect sizes, degrees of freedom and *P* value noted<br>*Give P values as exact values whenever suitable.*
☒	☐	For Bayesian analysis, information on the choice of priors and Markov chain Monte Carlo settings
☒	☐	For hierarchical and complex designs, identification of the appropriate level for tests and full reporting of outcomes
☐	☒	Estimates of effect sizes (e.g. Cohen's *d*, Pearson's *r*), indicating how they were calculated

Our web collection on statistics for biologists contains articles on many of the points above.

Software and code

Policy information about availability of computer code

Data collection	Varian GC 450 software, Agilent Technologies; Los Gatos Instruments software
Data analysis	R version 3.5.2, R Foundation for Statistical Computing; PAST version 3.20; Excel 2013, Microsoft Corporation

For manuscripts utilizing custom algorithms or software that are central to the research but not yet described in published literature, software must be made available to editors/reviewers. We strongly encourage code deposition in a community repository (e.g. GitHub). See the Nature Research guidelines for submitting code & software for further information.

Data

Policy information about availability of data

All manuscripts must include a data availability statement. This statement should provide the following information, where applicable:
- Accession codes, unique identifiers, or web links for publicly available datasets
- A list of figures that have associated raw data
- A description of any restrictions on data availability

Data used in this work are available from the corresponding author upon request and at the Smithsonian Institution figshare repository (https://smithsonian.figshare.com under the DOI 10.25573/serc.12855323

Field-specific reporting

Please select the one below that is the best fit for your research. If you are not sure, read the appropriate sections before making your selection.

☐ Life sciences ☐ Behavioural & social sciences ☒ Ecological, evolutionary & environmental sciences

Ecological, evolutionary & environmental sciences study design

All studies must disclose on these points even when the disclosure is negative.

Study description	Two so-called marsh-organ experiments were conducted to assess the effect of relative sea level (in interaction with other factors) on methane emissions. In Experiment 1, relative sea level, atmospheric [CO2], and N availability were manipulated in a full-factorial design. In Experiment 2, relative sea level and plant species were manipulated in a full-factorial design. Finally, data were compared to data from the adjacent field site.
Research sample	Gas samples taken from flux chambers (closed chambers), which represents a standard technique for the assessment of greenhouse-gas emissions from mesocosms or field plots.
Sampling strategy	Design and replication were based on previous full-factorial field experiments conducted at the Smithsonian Global Change Research Wetland, and replication of n = 3 was expected sufficient to detect main and interaction effects with large effect sizes (Langley and Megonigal 2010, Nature). Higher replication was not possible due to logistical constraints.
Data collection	Data were collected by Lillian Aoki, Peter Mueller, and Genevieve Noyce<br><br>For the mesocosm experiments, gas samples were taken from the headspace of flux chambers at regular intervals. Gas samples were analyzed using gas chromatography.<br>- Experiment 1: Varian 450 GC, Agilent Technologies, data were recorded by Varian software.<br>- Experiment 2: Shimadzu GC-14A, Shimadzu Corporation, data were recorded manually by Peter Mueller.<br>For the field measurements, CH4 emissions were measured using an ultraportable LGR greenhouse-gas analyzer, data were recorded using LGR software.
Timing and spatial scale	Experiment 1: Daily 12-14 July 2011 (first half of mesocosms) and 19-21 July 2011(second half of mesocosms).<br>Experiment 2: 06 Sep 2012<br>Spatial scale experiments: Field-based mesocosm set-up, all mesocosms situated in an area of approx. 3 x 15 m.<br>Field data: Monthly measurements from Jun-Sep 2019; spatial scale: marsh scale (i.e. approx. 300 x 300 m)
Data exclusions	Methane emissions were only measured from mesocosms with living plants. Extreme sea-level treatments caused die-off in some mesocosms, which were excluded from sampling. Only methane fluxes with linear R2-values > 0.8 were used. Compare Material and methods as well as original raw data.
Reproducibility	No attempts to repeat experiments
Randomization	Mesocosms were evenly distributed across 6 marsh organs. Allocation of mesocosms to treatments was randomized. Sampling within marsh organs was randomized. Sampling order of marsh organs was randomized, and marsh organ was included as random factor in the statistical analyses.
Blinding	No blinding was used. Blinding in field ecological studies is not a common practice and, in most cases, not feasible. The treatment to which single experimental units / mesocosms were exposed is obvious given the field experimental design.

Did the study involve field work? ☒ Yes ☐ No

Field work, collection and transport

Field conditions	Air temp. 27.5-34°C, no rain, low tide
Location	Smithsonian Global Change Research Wetland, Edgewater, Maryland, United States (38°530N, 76°330W).
Access and import/export	n/a, site is Smithsonian property
Disturbance	no disturbance

Reporting for specific materials, systems and methods

We require information from authors about some types of materials, experimental systems and methods used in many studies. Here, indicate whether each material, system or method listed is relevant to your study. If you are not sure if a list item applies to your research, read the appropriate section before selecting a response.

Materials & experimental systems

n/a	Involved in the study
☒	☐ Antibodies
☒	☐ Eukaryotic cell lines
☒	☐ Palaeontology
☒	☐ Animals and other organisms
☒	☐ Human research participants
☒	☐ Clinical data

Methods

n/a	Involved in the study
☒	☐ ChIP-seq
☒	☐ Flow cytometry
☒	☐ MRI-based neuroimaging
